# Multi-tissue metabolomic profiling reveals potential mechanisms of cocoon yield in silkworms (*Bombyx mori*) fed formula feed versus mulberry leaves

**DOI:** 10.3389/fmolb.2022.977047

**Published:** 2022-08-17

**Authors:** Xuehui Wu, Xuedong Chen, Aihong Ye, Jinru Cao, Ruimin He, Meiliang Pan, Feng Jin, Huanyan Ma, Wenlin Zhou

**Affiliations:** ^1^ Institute of Sericulture and Tea, Zhejiang Academy of Agricultural Sciences, Hangzhou, Zhejiang, China; ^2^ Shengzhou Mulsun Biotech Co., Ltd., Shengzhou, Zhejiang, China; ^3^ Zhejiang Provincial Agricultural Technology Extension and Service Center, Hangzhou, Zhejiang, China

**Keywords:** silkworm, metabolome profile, biomarker, formula feed, mulberry leaves

## Abstract

Use of formula feed (FF) for silkworms for all instars, has promoted transformation and progress in traditional sericulture. However, the cocoon yield of FF silkworms has failed to reach that of silkworms fed mulberry leaves (ML). The biological mechanisms underlying this phenomenon have not been well described. This study aimed to identify metabolic mechanisms and potential biomarkers relating to the poor cocoon yield of FF silkworms. In this study, silkworms received treatments of either ML (ML group) or FF (FF group) for all instars. At the 3rd day of the 5th instar, the midgut (MG), hemolymph (HL) and posterior silk gland (PSG) were collected for the metabolome profiles detection. The remaining silkworms were fed ML or FF until cocooning for investigation. The whole cocoon yield (WCY) was significantly higher in the FF group than the ML group (*p* < 0.05), whereas the cocoon shell weight (CSW) and cocoon shell rate (CSR) were significantly lower in the FF group (*p* < 0.05). A total of 845, 867 and 831 metabolites were qualified and quantified in the MG, HL and PSG of the FF silkworms, respectively. Correspondingly, 789, 833 and 730 metabolites were quantified in above three tissues of the ML group. Further, 230, 249 and 304 significantly different metabolites (SDMs) were identified in the MG, HL and PSG between the FF and ML group, respectively. Eleven metabolic pathways enriched by the SDMs were mutual among the three tissues. Among them, cysteine and methionine metabolism, arginine biosynthesis, and arginine and proline metabolism were the top three pathways with the highest impact value in the PSG. Six biomarkers were obtained through biomarker analysis and Pearson correlation calculation. Among them, homocitrulline, glycitein, valyl-threonine, propyl gallate and 3-amino-2,3-dihydrobenzoic acid were positively correlated with WCY, but negatively correlated with CSW and CSR (*p* < 0.05). An opposite correlation pattern was observed between 3-dimethylallyl-4-hydroxyphenylpyruvate and the three cocoon performance traits. Overall, three key metabolic pathways and six biomarkers associated with cocoon yield were interpreted, and should provide directions for formula feed optimization in factory-raised silkworms.

## 1 Introduction

The domesticated silkworm (*Bombyx mori*) is a Lepidoptera model organism that has been reared and domesticated on mulberry leaves (ML) for thousands of years (Tao et al., 2022). However, with continual optimization and changes in rural economic structure, Chinese sericulture is facing unprecedented challenges. Silkworm rearing on formula feed (FF), a method that originated in the middle of last century in Japan (Nair and Kumar, 2004), is becoming a major focus in the sericulture industry, because it alleviates season, climate, land and environmental restrictions (Kim et al., 2021). In 2019, rearing the silkworm variety “Zhong 2016×Ri 2016” on FF for all instars was successful in a commercial industry. However, the issue of poor cocoon yield in the FF silkworms remains unresolved, thus hindering sericulture.

It has been acknowledged that the three cocoon yield performance traits: the whole cocoon yield (WCY), the cocoon shell weight (CSW) and the cocoon shell rate (CSR) has drawn much attention in researches (Ruan et al., 2021; Chen et al., 2022), for they being closely related to sericulture profits. The WCY consists of CSW and the pupae weight, which can be utilized to evaluate the growth and development of the silkworm larva and its cocooning ability. The CSW is an indicator to assess the silk synthesis ability, while the CSR is used to evaluate the efficiency of nutrients to silk. However, there is no standard for which levels could be more desirable of these three performance traits in the production owing to the variety differences. For example, the cocoon shell weight of silkworm variety “Zhong 2016×Ri 2016” was more than 0.40 and 0.35 g in the ML and FF silkworms, respectively (Chen et al., 2022). In detail, the CSW and CSR of “Zhong 2016×Ri 2016” were decreased by 13.1 and 4.19% in the FF group, respectively (Chen et al., 2022).

Many studies have been performed to improve the cocoon yield performance of the FF silkworms to the greatest extent possible (Zhang et al., 2020; Kim et al., 2021; Yamamoto et al., 2021). Most have focused on optimizing the FF composition (Kim et al., 2021; Yamamoto et al., 2021) and silkworm variety breeding (Zhang et al., 2020). However, the mechanisms underlying the poor cocoon yield of the FF silkworms have not been well documented. With the rapid development of systems biology, omics methods have been widely used in livestock studies, including the sericulture field (Dong et al., 2018; Fang et al., 2020; Ge et al., 2020; Xiao et al., 2020; Tao et al., 2022). Researchers have explored the molecular differences between the FF and ML silkworms (Zhou et al., 2008; Dong et al., 2017; Dong et al., 2018; Lamberti et al., 2019; Qin et al., 2020; Tao et al., 2022). For instance, the intestinal microbiota diversity was reported to be higher in the ML silkworms than the FF silkworms (Dong et al., 2018). Lamberti et al. (2019) found five differentially expressed proteins associated with diet type through comparative proteomics. Furthermore, the content of pupa proteins in the FF silkworms was markedly higher than that in traditional ML silkworms as reported (Lamberti et al., 2019).

Metabolomics can qualify and quantify thousands of metabolites, and describe the specific metabolomic profiles of certain tissues or biofluids at specific time points in organisms (Saoi and Britz-McKibbin, 2021). Currently used detection platforms are based on gas chromatography-mass spectrometry, liquid chromatography-mass spectrometry (LC-MS) and nuclear magnetic resonance, each of which has its own merits. LC-MS aids in identifying metabolites with high polarity and high formula weight, and can quantify and qualify more metabolites than other platforms (Tao et al., 2022). Metabolomics has been applied in exploring the metabolic profiles of silkworms subjected to two rearing methods (FF versus ML) (Dong et al., 2017; Qin et al., 2020; Tao et al., 2022). However, previous studies have focused mainly on metabolic changes in a single tissue; therefore, the results cannot fully reflect systematic variations of the whole organism. Consequently, multi-tissue studies on modern sericultural research are necessary. Hemolymph (HL), the transporter of nutrients and intermediates, reflects changes in the whole body (Tao et al., 2022). Zhou et al. (2015) have reported that changes in the HL metabolome are closely associated with dietary composition, cell membrane biosynthesis, and protein and energy metabolism. However, the non-specific characteristic of the HL restricts the explain validity to some issues. The midgut (MG) plays a crucial role in nutrient digestion and absorption (Shen et al., 2022), and thus warrants further nutrition metabolism studies. Posterior silk gland (PSG) is the locus of fibroin production (Shigematsu and Koyasako, 1962), which is closely associated with various cocoon performance traits. Consequently, integrating the metabolomic profiles of the three aforementioned tissues should provide systematic knowledge in silkworm nutritional biology.

This study aimed to identify mechanisms and biomarkers relating to poor cocoon yield in silkworms reared on formula feed for all instars, by integrating systematic metabolomic profiling.

## 2 Materials and methods

### 2.1 Animals and rearing

The *B. mori* variety “Zhong 2016×Ri 2016,” provided by the Zhejiang Silkworm Egg Quality Inspection Station (Hangzhou, China), was studied. Three thousand silkworms were divided into six groups and were randomly allocated to receive two diet treatments: formula feed (FF group) and fresh mulberry leaves (ML group) for all instars. The FF silkworms were fed once from the newly hatched period to the 2nd instar, and were fed one, one and two times in the 3rd, 4th and 5th instars, respectively. The ML group was fed three times at 7:00 a.m., 13:00 p.m. and 19:00 p.m. per day during five instar periods.

All silkworms were reared in an intelligent and independent room with relatively optimal room temperature and humidity, according to the different requirements of each instar. All remaining experimental silkworms were reared to cocoon stage for further cocoon yield performance investigation, including the WCY, CSW and CSR.

### 2.2 Diet preparation

The ingredients of the formula feed were based on a report by Dong et al. (2017). The formula contained 33% mulberry leaf powder for silkworms in the 1st -3rd instars, whereas 4th to 5th instar silkworms were fed formula feed containing 25% mulberry leaves. The formula feed powder was mixed with 1.85× (w/w) sterile water. The mixture was placed in a storage bag, pressed to a thickness of approximately 1.00 cm and heated for 50 min at 100°C after sealing. After heating, the formula feed mixture was naturally cooled and stored at 4°C for feeding trials.

Mulberry leaves were picked from the Nongsang No.14 mulberry variety every morning and were stored at 4°C to maintain freshness. Nongsang No.14 with good mulberry leaf quality was provided by Shaoxing Dayu Silkworm Egg Production Co., Ltd. (Shaoxing, China).

### 2.3 Sample collection and preparation

#### 2.3.1 Sample collection

The 3rd day of the 5th instar is the most active time point of silkworm larval metabolism (Dong et al., 2017). MG, HL and PSG were collected on ice at the 5th instar after feeding for 72 h. HL was gathered by cutting the anal horns of the silkworm larvae. Those silkworm larvae were fixed and cut in a dissecting pan to obtain the experimental MG and PSG with specialized surgical nippers and scissors. Groups of six silkworm tissues were combined into one sample replicate. A total of 12 samples per tissue were analyzed, consisting of six male and six female replicates. To terminate the metabolic activity of each tissue, every sample was frozen in liquid nitrogen as soon as possible and was stored at −80°C for subsequent metabolomic profiling analysis.

#### 2.3.2 Sample preparation for metabolite detection

Sample preparation, including thawing and following procedures before detection, was performed on ice.

##### 2.3.2.1 HL

On the basis of the readily oxidizable property of silkworm HL, nitrogen oxides were inflated to the refrigerated HL sample for 5 s after the experimental samples were removed from −80°C storage. The thawed HL was vortexed for 10 s, and 50 μL of sample was added to an Eppendorf (EP) tube containing 300 μL pure methanol with 0.1% butylated hydroxytoluene and internal standard extract (1 ppm: [^2^H3]-L-carnitine HCl, 4-fluoro-L-α-phenylglycine, L-phenylalanine (2–13C, 99%), L-2-chlorophenylalanine, [^2^H5]-kynurenic acid, [^2^H5]-hippuric acid, [^2^H5]-phenoxy acetic acid). The above mixtures were vortexed for 3 min and centrifuged for 4 min at 12,000 rpm (r = 0.15 m) and 4°C. Subsequently, 200 μL supernatant was transferred to a new EP tube to silence for 30 min in −20°C. Then, the above supernatants were centrifuged at 12,000 rpm for 10 min at 4°C. Finally, 150 μL supernatant was transferred into a brown injection bottle and stored at −20°C for UHPLC-MS/MS detection.

##### 2.3.2.2 MG and PSG

Tissue samples were thawed, and approximately 50 mg was cut and placed into a clean 1.5 ml EP tube. Steel balls were added to the EP tubes, and samples were homogenized at 30 Hz for 3 min. The mixture was vortexed for 5 min after 1 ml 70% methanol with the aforementioned internal standard extract was added. The mixture was placed on ice for 10 min before centrifugation (4°C, 12,000 rpm, 10 min). Subsequently, 400 μL supernatant was transferred into a new EP tube and stored overnight at −20°C. Finally, the supernatant was centrifuged for 3 min at 12,000 rpm and 4°C, and 200 μL supernatant was transferred to an injection bottle for UHPLC-MS/MS detection.

Of note, after every ten samples. 10 μL of the prepared sample supernatants of each tissue was combined into a mixed sample, which served as the QC for UHPLC-MS/MS detection, to evaluate the manual accuracy, instrument stability and technology replicability.

### 2.4 UHPLC-MS/MS detection and analysis

#### 2.4.1 Compound detection by UHPLC-MS/MS

The above sample extracts were analyzed with a UHPLC (ExionLC FF, https://sciex.com.cn/)-electrospray ionization (ESI)-MS/MS system (QTRAP^®^ System, https://sciex.com/).

The UHPLC conditions were as follows: Waters ACQUITY UPLC HSS T3 C18 column (1.8 µm, 2.1 mm × 100 mm), A phase: ultrapure water (0.1% formic acid), B phase: acetonitrile (0.1% formic acid), column temperature: 40°C, injection volume: 2 μL. The UHPLC gradient elution was conducted as follows: 95:5 V/V (A phase/B phase) at 0 min, 10:90 V/V at 10.0 min, 10:90 V/V at 11.0 min, 95:5 V/V at 11.1 min and 95:5 V/V at 14.0 min.

The QTRAP^®^ LC-MS/MS system equipped with an ESI Turbo Ion-Spray interface was used to acquire the linear ion trap and triple quadrupole (QQQ) scans. This system was controlled by Sciex Analyst 1.6.3 software (https://sciex.com.cn/), and was operated in positive and negative ion mode. The ESI parameters were set as follows: source temperature: 500°C; ion spray voltage: 5500 V (positive) to 4500 V (negative); ion source gas I, gas II and curtain gas set at 55.0, 60.0 and 25.0 psi, respectively; collision-activated dissociation parameter set high. Equipment tuning and mass calibration were performed with 10 and 100 μmol/L polypropylene glycol solutions in QQQ and linear ion trap modes, respectively. In QQQ scans, each ion pair was detected on the basis of the optimal declustering potential and collision energy (Chen et al., 2013).

#### 2.4.2 Identification and quantification of metabolites

Compound identification was based on the metware database, retention time, macroion and precursor ion information, and secondary spectrum data of specific compounds.

Multiple reaction monitoring of the QQQ system was used to quantify the compounds detected. The areas under each chromatographic peak were calculated and represented the relative content of the detected compound in MultiQuant software (https://sciex.com.cn/). On the basis of the retention time and peak pattern information of the detected compounds, integral correction in different samples was performed to ensure the qualification and quantification accuracy (Fraga et al., 2010). Metabolites were defined as detected if they were present in more than 50% of the QC samples. Similarly, metabolites were considered to exist in a given group if they were detected in at least 50% of the samples in the group.

#### 2.4.3 Unique and mutually present metabolites in the MG, HL and PSG

To identify the metabolic similarities and differences among the MG, HL and PSG, we analyzed the unique and mutual metabolites in different tissues in this study.

### 2.5 Statistical analysis

#### 2.5.1 Significantly different metabolites identification between the ML and FF group

Metaboanalyst 5.0 (https://www.metaboanalyst.ca/) was used to perform multivariate data analysis (MVDA) under the log transformation of data and auto scaling mode (Pang et al., 2021). Sample normalization was set to “normalized by sum”. MVDA, as conducted in this study, included PCA and OPLS-DA. The PCA model was used to observe the global distribution of experimental samples. OPLS-DA was performed to identify the metabolomic differences among treatments, particularly in distinguishing different metabolites. Missing value estimation was performed by default. In detail, missing values were replaced by one-fifth of the minimum positive values in corresponding variables.

The VIP >1.00, FC > 2.00 or < −2.00 and FDR <0.05 were the criteria used to define SDMs, which were further identified, validated and classified in the KEGG and HMDB. Mutual and unique SDMs were analyzed and shown in Venn diagrams. Furthermore, the ∣log_2_FC∣ was the index used to identify the top 15 up-regulated and the top 15 down-regulated SDMs in three tissues.

#### 2.5.2 Pathway analysis based on the significantly different metabolites

Pathway analysis was conducted in Metaboanalyst 5.0, with the fruit fly (*Drosophila melanogaster*) library applied in this step. Sample normalization, data transformation and data scaling was set “Normalization by sum”, “Log transformation” and “Data scaling”, respectively. In this study, the impact value (IPV) > 0.100 was set as the cutoff for relevance (Sun et al., 2015; Wu et al., 2019).

#### 2.5.3 Biomarker and correlation analysis

Biomarker analysis was conducted in Metaboanalyst 5.0 with the relative concentrations of metabolites detected. To improve the reliability of results, metabolites with level B were discarded in this procedure. SDMs with an AUC = 1 served as the preselected biomarkers to explain and indicate the metabolic differences between the FF and ML silkworms across the three tissues.

Preselected biomarkers mutually present among the MG, HL and PSG were chosen for correlation analysis with the cocoon yield performance traits (WCY, CSW and CSR). Correlation analysis was conducted in the R package (https://www.r-project.org/) using Pearson coefficient.

#### 2.5.4 Performance traits analysis

In sericulture production, male and female silkworms are fed together in nearly all silkworm varieties. Normally, the proportion of male and female silkworms bred is 1:1. So equal number of male and female silkworms were collected together to be consistent with the production. In addition, for the three cocoon performance traits in this study, there was no interaction between the diet and sex in the statistical model. Thus, sex was not included in the model. In detail, the SAS MIXED model and the univariate statistical analysis was used to analyze the WCY, CSW and CSR between the FF and ML group. Diets was set as the only factor in the statistical model. *p* < 0.05 was defined as the significance threshold, and *p* < 0.01 was regarded as an extremely significant difference.

## 3 Results

### 3.1 Cocoon yield performance

**FIGURE 1:**
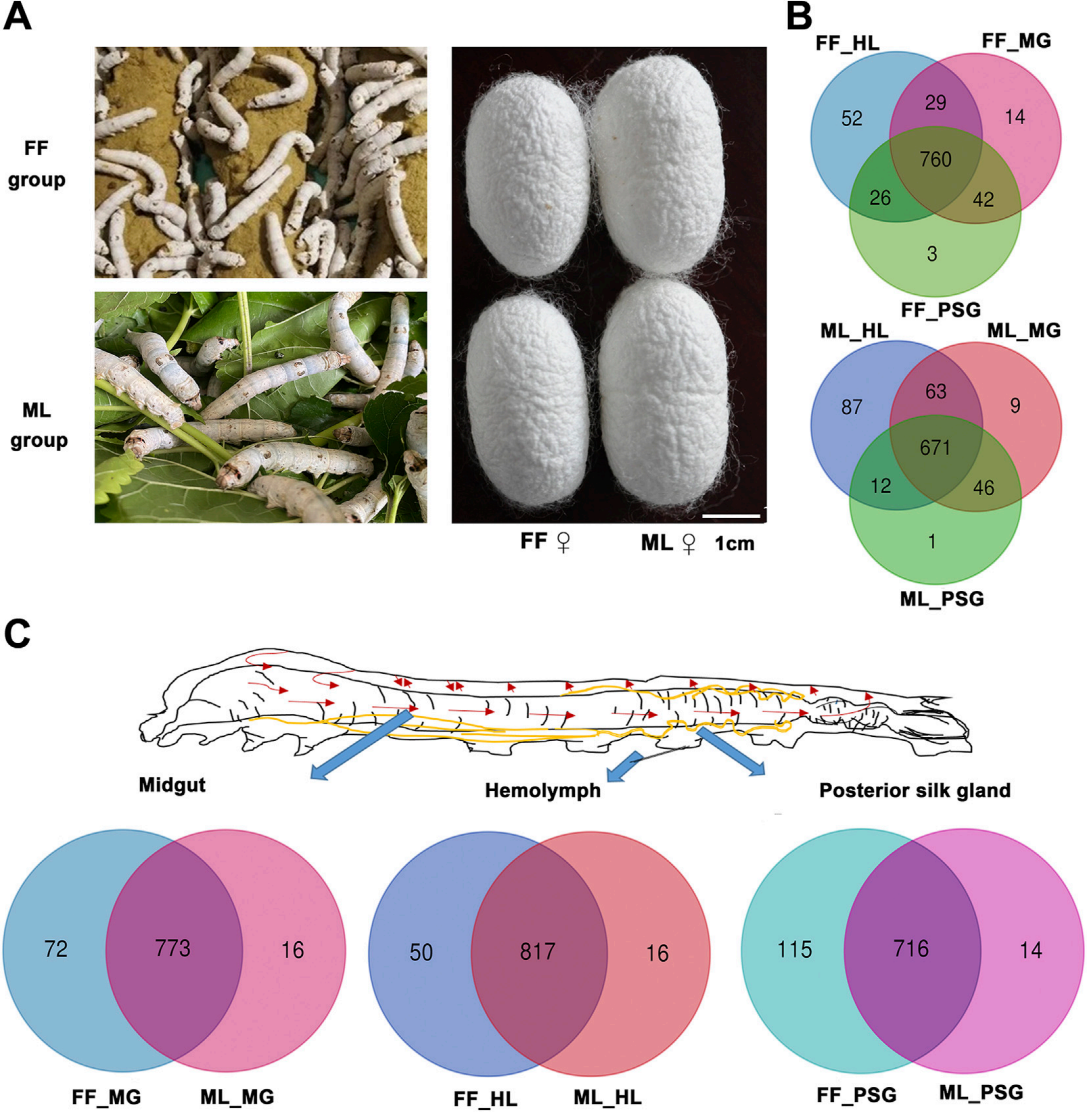
Metabolites distribution in the hemolymph (HL), midgut (MG) and posterior silk gland (PSG) between the FF and ML silkworms. **(A)** Silkworms fed formula feed (FF) or mulberry leaves (ML), and corresponding cocoons. **(B)** Metabolites distribution among three tissues in the FF (up) and ML (down) silkworms. **(C)** Unique and mutual metabolites identified in different tissues in the FF and ML groups.

In this study, WCY, CSW and CSR, typical cocoon yield performance traits were investigated and analyzed between the FF and ML silkworms (Figure 1A; Table 1). The WCY was 10.6% higher in the FF group than the ML group (*p* < 0.05), whereas the CSW and CSR of the FF group were 11.7 and 20.5% lower than the ML group, respectively (*p* < 0.05). Besides, the pupa of FF group was 17.1% heavier than those of ML group (*p* < 0.05).

**TABLE 1:** The cocoon yield performance of silkworms reared on formula feed or mulberry leaves.

Treat^a^	WCY^b^, g	CSW^c^, g	CSR^d^, %	PW^e^, g
FF group	2.08 ± 0.114^a^	0.371 ± 0.0228^b^	17.8 ± 0.34^b^	1.71 ± 0.085^a^
ML group	1.88 ± 0.016^b^	0.420 ± 0.0126^a^	22.4 ± 0.62^a^	1.46 ± 0.015^b^

a FF group, silkworms reared on formula feed; ML group, silkworms reared on mulberry leaves.

b WCY, whole cocoon yield.

c CSW, cocoon shell weight.

d CSR, cocoon shell rate.

e PW, pupae weight.

Data are presented as mean ± standard deviation.

Different letters within a column indicate significant differences between FF and ML groups (*p* < 0.05).

### 3.2 UHPLC-MS/MS detection and analysis

#### 3.2.1 Metabolite identification and quantification

The total ion chromatogram information under positive and negative ion modes are shown in Supplementary Figure S1. A total of 1,033 compounds were identified by ultra-high performance LC-MS/MS (UHPLC-MS/MS). After filtering, 927 metabolites were finally qualified. The results of compound identification and quantification are described in Supplementary Table S1. Different letters (A or B) in the level column indicate different levels of identified metabolites (Supplementary Table S1), which were associated with subsequent biomarker analysis. Level A indicates that the information on two ion fragments was consistent with the metware database, whereas level B indicates that only one ion fragment was consistent.

#### 3.2.2 Metabolite distribution

The number of metabolites detected in three tissues of silkworms fed the two diets are shown in Figure 1B and Figure 1C. A total of 845, 867 and 831 metabolites were identified in the MG, HL and PSG of the FF silkworms, respectively. Correspondingly, 789, 833 and 730 metabolites were detected in the MG, HL and PSG of the FF group, respectively.

The metabolite distribution in the analyzed tissues in the FF and ML silkworms is depicted in Figure 1B. A total of 760 metabolites were mutual in the MG, HL and PSG of the FF group, whereas 671 metabolites were mutual in the corresponding tissues of the ML silkworms. A total of 52 and 87 specific metabolites were detected in the HL of the FF and ML groups, respectively. Only 14 and three metabolites were specific in the MG and PSG of the FF group. Similarly, nine metabolites and one metabolite were specific in the MG and PSG of the ML group, respectively.

Similarly, the metabolite distributions (Figure 1C) indicated that 773, 817 and 716 metabolites were mutual in the MG, HL and PSG of the FF and ML groups. The numbers of specific metabolites in the FF group were 72, 50 and 115, and those in the ML group were 16, 16 and 14 in the MG, HL and PSG, respectively.

#### 3.2.3 Multivariate statistical comparison of metabolites between the FF and ML groups

**FIGURE 2:**
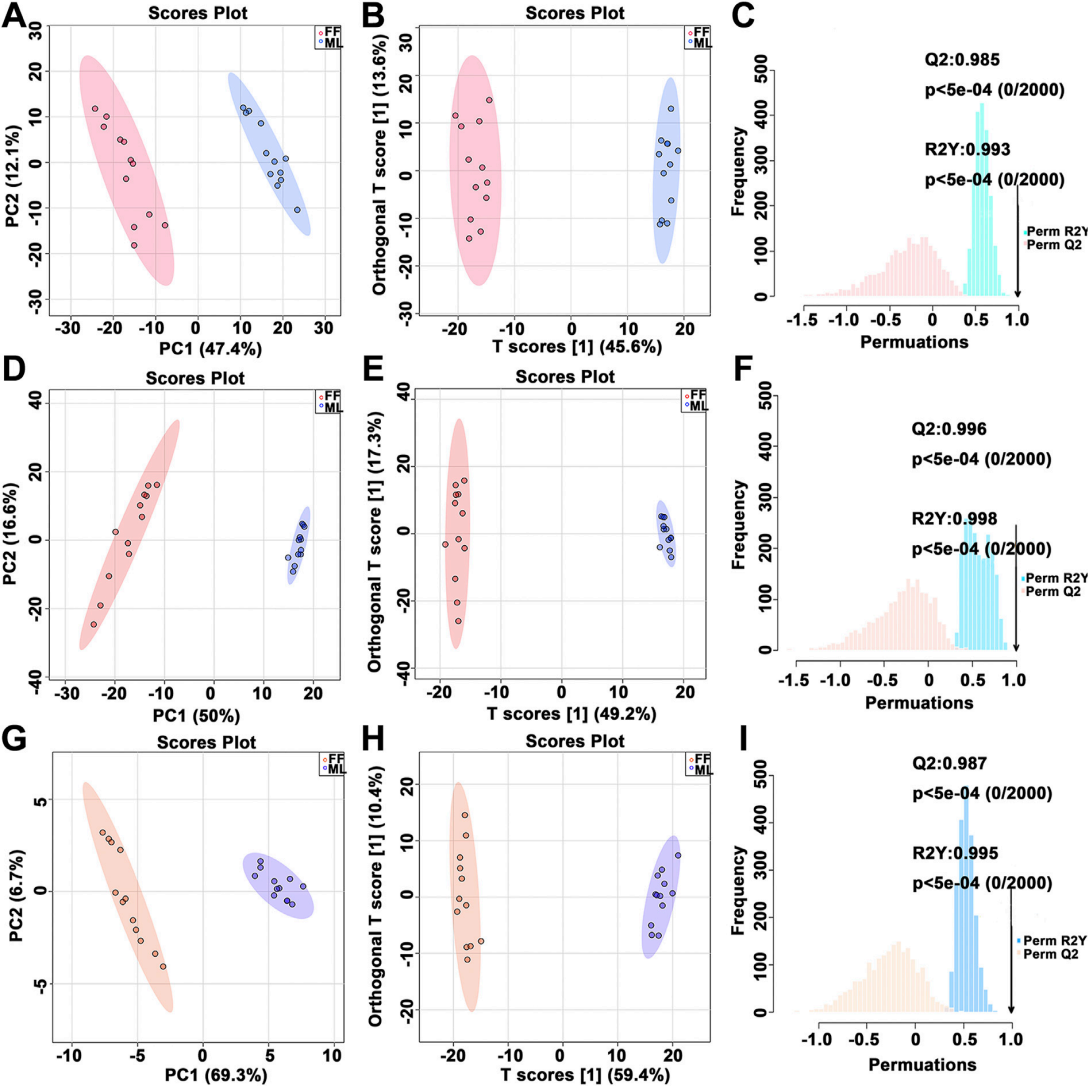
PCA score plot, OPLS-DA score plot and corresponding permutation test results of the metabolite profiles. PCA, principal component analysis; OPLS-DA, orthogonal partial least squares discriminant analysis. PCA score plot **(A,D,G)**, OPLS-DA score plot **(B,E,H)** and corresponding permutation test results **(C,F,I)** were derived from the metabolite profiles of the midgut (MG, **(A–C)**, hemolymph (HL, **(D–F)** and posterior silk gland (PSG, **(G–I)**. Red dots represent silkworms fed formula feed (FF), and blue dots represent silkworms fed mulberry leaves (ML).

Figure 2 shows the multivariate statistical analysis (MVDA) results of the metabolic profiling between the FF and ML silkworms in the MG (A-C), HL (D-F) and PSG (**G-I**). For the MG metabolic profile, a significant separation was observed between the FF and ML groups in the principal component analysis (PCA) score plot. For the HL and PSG, a similar pattern was demonstrated. No outliers were beyond the 95% Hotelling’s T-squared ellipse in all PCA score plots. The PCA score plot contained quality control (QC) samples is shown in Supplementary Figure S2 (**A**-MG, B-HL, **C**-PSG). All QC samples clustered together and was separately from the FF and ML samples (Supplementary Figure S2). The orthogonal partial least squares discriminant analysis (OPLS-DA) score plot also showed two clearly distinct clusters, with no samples exceeding the 95% ellipse. Furthermore, 2000 permutation tests indicated that the OPLS-DA model was suitable for significantly different metabolites (SDMs) identification (MG: R2Y = 0.993 and Q2 = 0.985, HL: R2Y = 0.998 and Q2 = 0.996 and PSG: R2Y = 0.995 and Q2 = 0.987).

#### 3.2.4 Significantly different metabolite identification between the FF and ML silkworms

**FIGURE 3:**
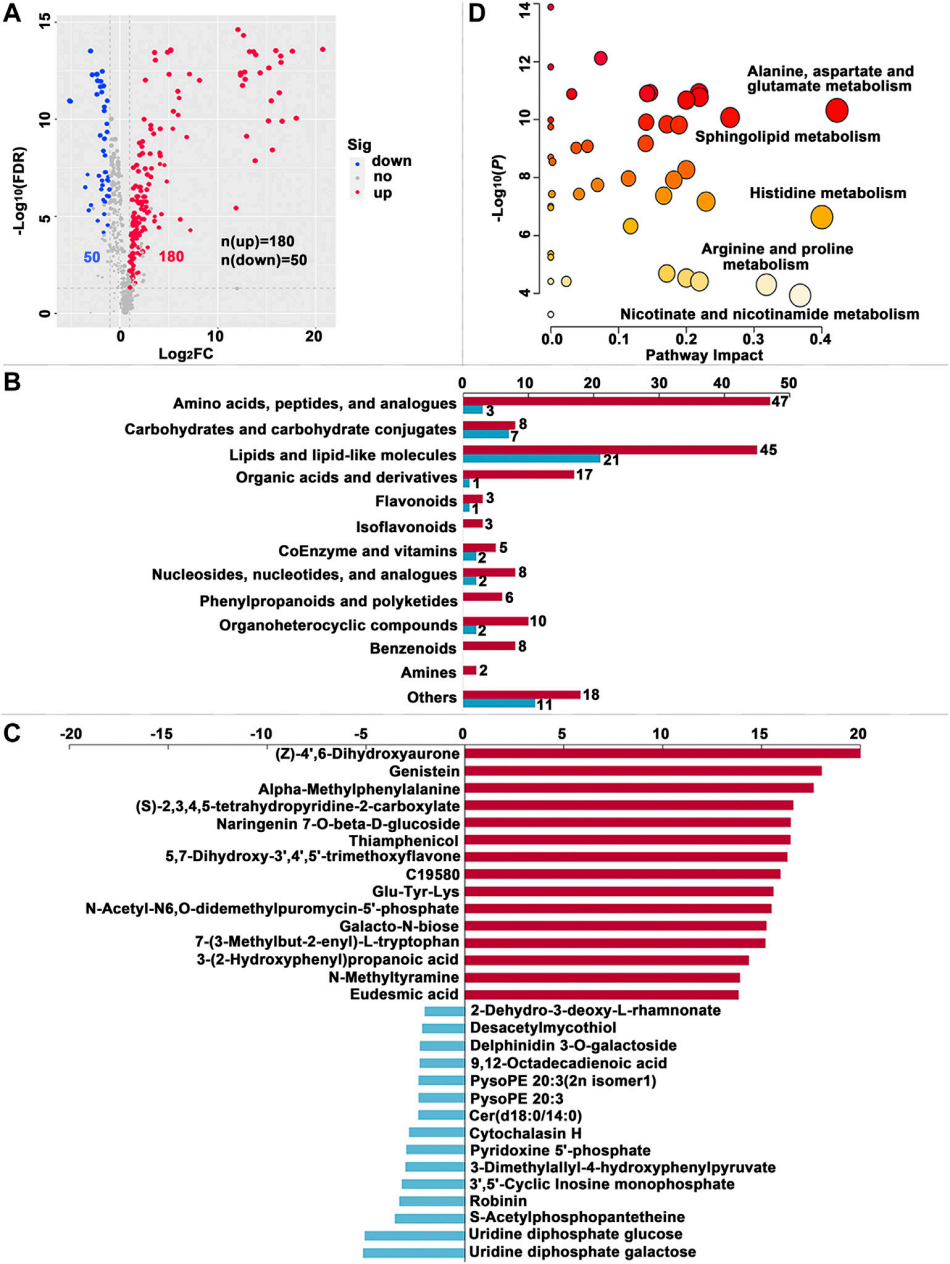
Significantly different metabolites (SDMs) and enriched pathways relating to different diets in themidgut (MG). **(A)** Volcano plots of metabolites for the formula feed (FF) and the mulberry leaves (ML) silkworms in the MG. The red dot represents the metabolite is significantly higher in the FF silkworms, and the blue dot indicates a higher abundant metabolite in theML silkworms. The dot size represents the VIP value. **(B)** SDMs classification and variation between the FF and ML group. The blue and red bar indicate the number of downregulated and upregulated metabolites in theMG of FF group, respectively. **(C)** The top 15 upregulated SDMs and downregulated SDMs in the MG of the FF silkworms. **(D)** Metabolic pathways enriched by the SDMs between the FF and ML groups in the MG. C19580, alpha-[3-[(Hydroxymethyl)nitrosoamino]propyl]-3-pyridinemethanol.

**FIGURE 4:**
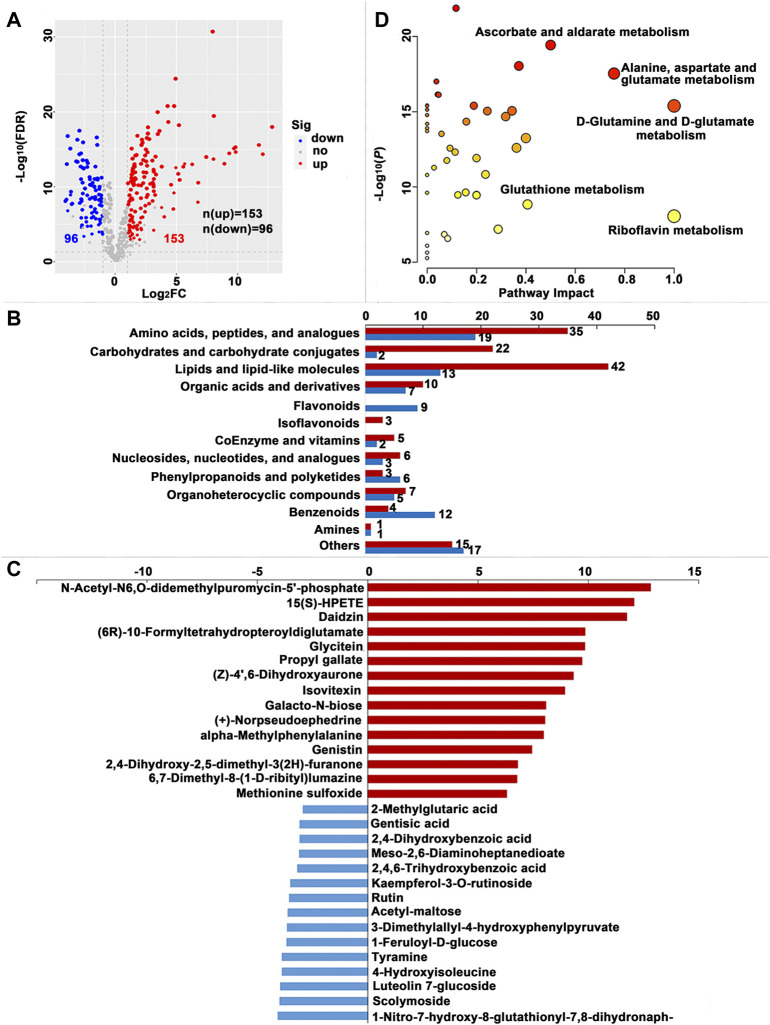
Significantly different metabolites (SDMs) and enriched pathways relating to different diets in the hemolymph (HL). **(A)** Volcano plots of metabolites for the formula feed (FF) and the mulberry leaves (ML) silkworms in the HL. The red dot represents the metabolite is significantly higher in the FF silkworms, and the blue dot indicates a higher abundant metabolite in the ML silkworms. The dot size represents the VIP value. **(B)** SDMs classification and variation between the FF and ML group. The blue and red bar indicate the number of downregulated and upregulated metabolites in the HL of the FF group, respectively. **(C)** The top 15 upregulated SDMs and downregulated SDMs in the HL of the FF silkworms. **(D)** Metabolic pathways enriched by the SDMs between the FF and ML groups in the HL.

**FIGURE 5:**
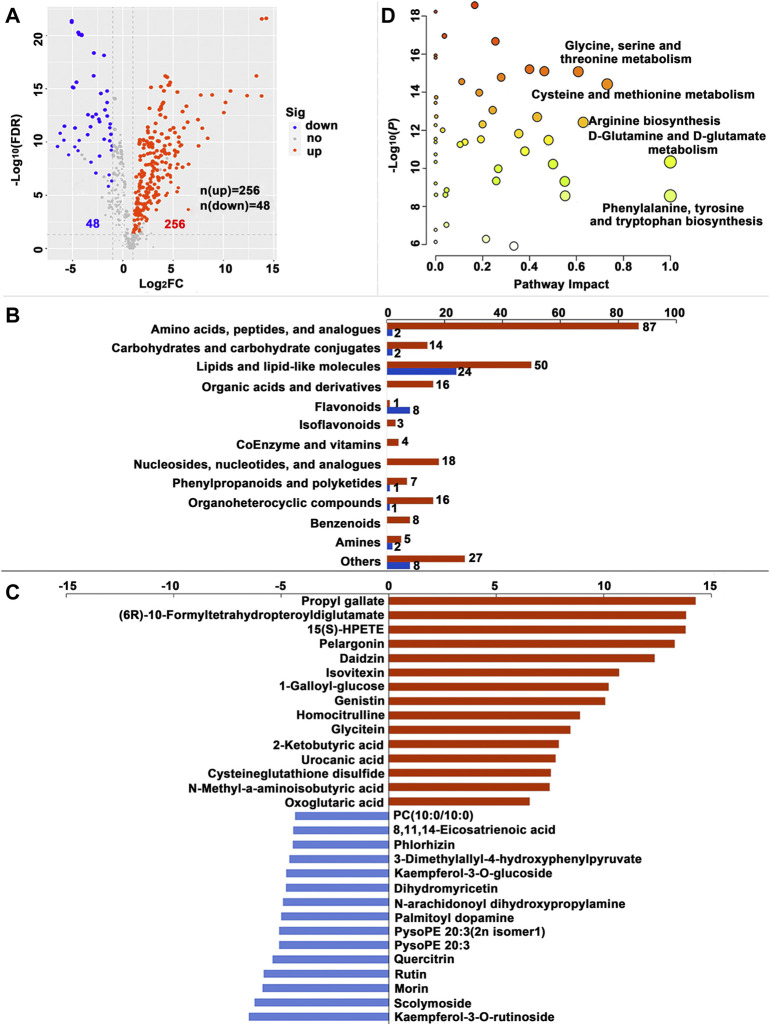
Significantly different metabolites (SDMs) and enriched pathways relating to different diets in the posterior silk gland (PSG). **(A)** Volcano plots of metabolites for the formula feed (FF) and the mulberry leaves (ML) silkworms in the PSG. The red dot represents the metabolite is significantly higher in the FF silkworms, and the blue dot indicates a higher abundant metabolite in the ML silkworms. The dot size represents the VIP value. **(B)** SDMs classification and variation between the FF and ML group. The blue and red bar indicate the number of downregulated and upregulated metabolites in the PSG of the FF group, respectively. **(C)** The top 15 upregulated SDMs and downregulated SDMs in the PSG of the FF silkworms. **(D)** Metabolic pathways enriched by the SDMs between the FF and ML groups in the PSG.

A total of 230, 249 and 304 SDMs (Variable importance for the projection (VIP) > 1.00, fold change (FC) > 2.00 or FC < -2.00, false discovery rate (FDR) < 0.05) were identified in the MG, HL and PSG, respectively, between the FF and ML groups (Supplementary Table S2). In the volcano plot (Figure 3A, Figure 4A, Figure 5A), the upregulated metabolites in the FF silkworms are in red, whereas downregulated metabolites are in blue. A total of 180, 153 and 256 upregulated SDMs were found in the MG, HL and PSG, respectively. In contrast, the numbers of downregulated SDMs of the MG, HL and PSG were 50, 96 and 48, respectively. The above upregulated and downregulated SDMs were categorized and shown in Figure 3B, Figure 4B and Figure 5B, respectively. In the MG metabolic profiling, the class of amino acids, peptides, and analogues; carbohydrates and carbohydrate conjugates; lipids and lipid-like molecules; and coenzymes and vitamins accounted for 50, 15, 66 and 7, respectively. In the HL, 54, 24, 55 and 7 SDMs were classified as amino acids, peptides, and analogues; carbohydrates and carbohydrate conjugates; lipids and lipid-like molecules and coenzymes and vitamins, respectively. In the metabolic profiles of the PSG, a large proportion of SDMs was classified as amino acids and their derivatives (n = 89) and lipids and lipid-like molecules (n = 74). Furthermore, carbohydrates and carbohydrate conjugates, and coenzymes and vitamins accounted for 16 and 4, respectively.

The top 15 upregulated SDMs and top 15 downregulated SDMs of the three tissues with the most significant differences (∣log_2_FC∣) are displayed in Figure 3C, Figure 4C and Figure 5C, respectively. Among them, 3-dimethylallyl-4-hydroxyphenylpyruvate was the only top 15 downregulated SDM mutually among the three tissues.

The mutual and specific SDM distribution results among the MG, HL and PSG are depicted in Supplementary Figure S3. A total of 60 SDMs were mutually found in the three tissues (Supplementary Table S3). The numbers of specific SDMs of the MG, HL and PSG were 75, 116 and 144, respectively.

#### 3.2.5 Identification of metabolic pathways related to SDMs

Metabolic pathways were enriched by the SDMs between the FF and ML groups among three tissues (Figure 3D, Figure 4D and Figure 5D, respectively.). In the MG, 23 metabolic pathways were enriched by the 230 SDMs, among which alanine, aspartate and glutamate metabolism (IPV = 0.422); histidine metabolism (IPV = 0.400); nicotinate and nicotinamide metabolism (IPV = 0.368); arginine, proline metabolism (IPV = 0.318); and sphingolipid metabolism (IPV = 0.265) were the top five enriched pathways (Supplementary Table S4). The SDMs of the HL were mainly enriched in 22 metabolic pathways (impact value (IPV) > 0.100). D-glutamine and D-glutamate metabolism (IPV = 1); riboflavin metabolism (IPV = 1.00); alanine, aspartate and glutamate metabolism (IPV = 0.757); ascorbate and aldarate metabolism (IPV = 0.500); and glutathione metabolism (IPV = 0.407) were the top five affected pathways (Supplementary Table S4). A total of 28 metabolic pathways were enriched in the SDMs of the PSG, among which D-glutamine and D-glutamate metabolism (IPV = 1.00); phenylalanine, tyrosine and tryptophan biosynthesis (IPV = 1.00); cysteine and methionine metabolism (IPV = 0.731); arginine biosynthesis (IPV = 0.629); and glycine, serine and threonine metabolism (IPV = 0.608) were the top five enriched pathways. Furthermore, phenylalanine metabolism (IPV = 0.552); glutathione metabolism (IPV = 0.550); and ascorbate and aldarate metabolism (IPV = 0.500) also showed high impact values (IPV ≥0.500, Supplementary Table S4).

**FIGURE 6:**
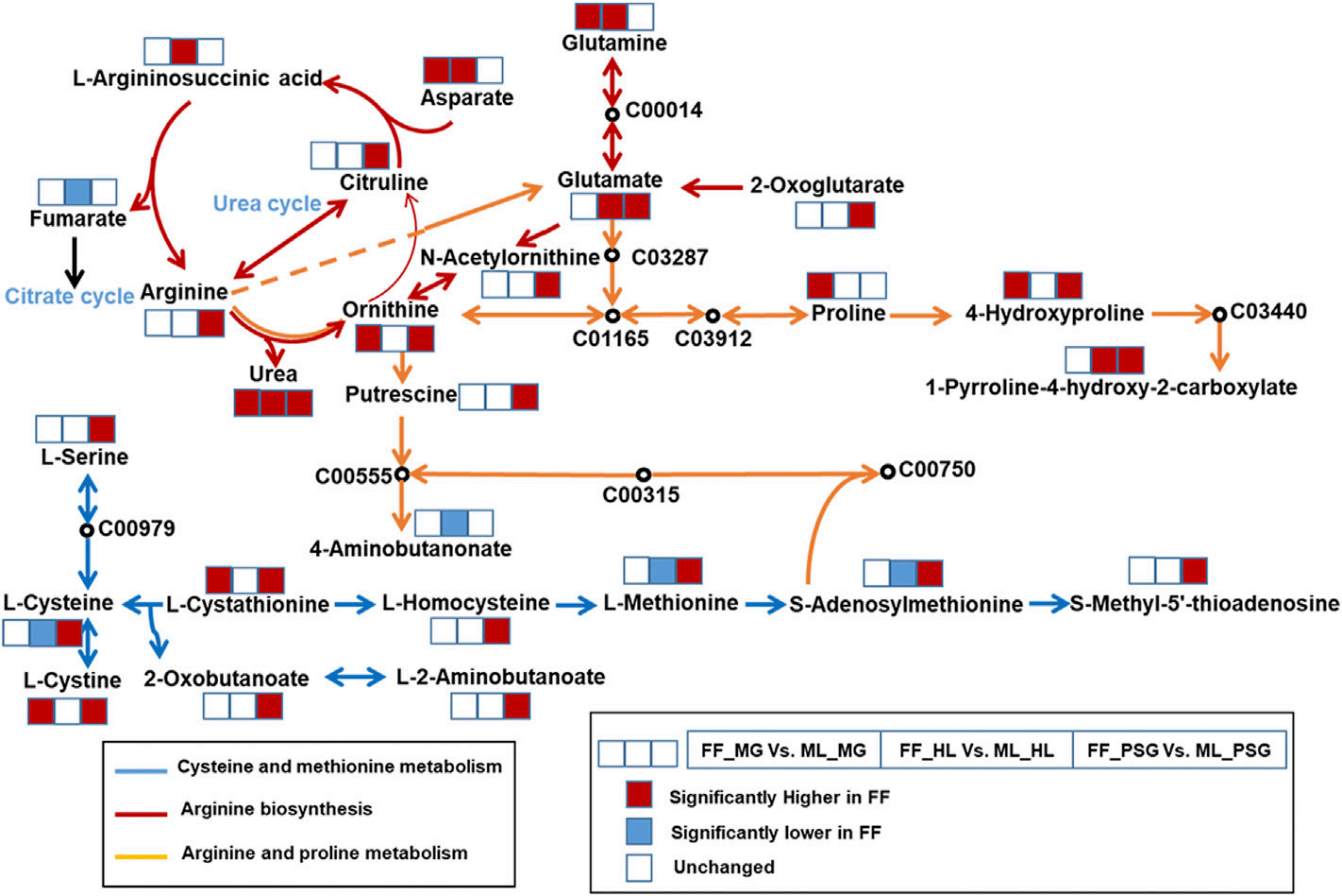
The integrated mutual metabolic pathways in the midgut (MG), hemolymph (HL) and posterior silk gland (PSG) of the formula feed (FF) silkworms. The blue, red and yellow line represented different metabolic pathways. The KEGG ID represented those were not detected or were not significantly different between the FF and mulberry leaves (ML) fed silkworms. The three squares with colors showed the different trends of metabolites in the MG, HL and PSG, respectively. The red, blue and blank squares represented the metabolites were significantly higher, lower or unchanged in the FF group, respectively.

A total of 11 mutual pathways were enriched in the SDMs across the three tissues. Among them, the top three mutual pathways with the highest IPV in the PSG were listed as follows, which was integrated and depicted in Figure 6. Cysteine and methionine metabolism (IPV = 0.731) was enriched by ten SDMs in the PSG: S-methyl-5′-thioadenosine (Log_2_FC = 2.02), S-adenosylmethionine (Log_2_FC = 3.15), L-cystathionine (Log_2_FC = 2.13), L-serine (Log_2_FC = 2.39), L-homocysteine (Log_2_FC = 2.74), L-methionine (Log_2_FC = 1.29), L-cystine (Log_2_FC = 3.46), L-cysteine (Log_2_FC = 2.98), L-2-aminobutanoate (Log_2_FC = 1.69) and 2-Oxobutanoate (Log_2_FC = 7.89). Arginine biosynthesis, with an IPV of 0.629, was enriched by the SDMs: L-glutamate (Log_2_FC = 4.09), L-arginine (Log_2_FC = 1.71), N-acetylornithine (Log_2_FC = 1.78), L-citrulline (Log_2_FC = 2.51), 2-oxoglutarate (Log_2_FC = 6.54), L-ornithine (Log_2_FC = 2.83) and urea (Log_2_FC = 5.43). Arginine and proline metabolism (IPV = 0.463) was enriched by the SDMs: L-arginine (Log_2_FC = 1.71), putrescine (Log_2_FC = 1.26), S-adenosylmethionine (Log_2_FC = 3.16), L-glutamate (Log_2_FC = 4.09), L-ornithine (Log_2_FC = 2.83), 4-hydroxyproline (Log_2_FC = 4.41) and 1-pyrroline-4-hydroxy-2-carboxylate (Log_2_FC = 2.32).

#### 3.2.6 Biomarkers associated with cocoon yield performance traits

A total of 722 metabolites with level A were used to perform biomarker analysis. Together with the differential trend of each metabolite (the selected metabolites are the SDMs in Results Section), 93, 171 and 163 preselected biomarkers (area under the curve (AUC) = 1) were obtained in the MG, HL and PSG, respectively (Supplementary Table S5). Among them, a total of 24 metabolites could serve as mutual preselected biomarkers across the three tissues (Supplementary Table S6).

**FIGURE 7:**
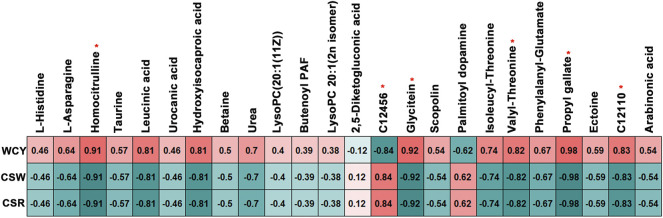
Correlation results between the cocoon yield performance traits and the six biomarkers identified. The red “∗” indicates biomarkers which was significantly correlated with the cocoon yield performance traits. WCY, whole cocoon yield; CSW, cocoon shell weight; CSR, cocoon shell rate. C12456, 3-Dimethylallyl-4-hydroxyphenylpyruvate; C12110, 3-Amino-2,3-dihydrobenzoic acid.

The correlation analysis indicated that six mutual preselected biomarkers were significantly associated with the WCY, CSW and CSR (*p* < 0.05, Figure 7). Among them, homocitrulline, glycitein, valyl-threonine, propyl gallate and 3-amino-2,3-dihydrobenzoic acid were positively correlated with WCY, but were negatively correlated with CSW and CSR. In contrast, an opposite correlation pattern was observed between 3-dimethylallyl-4-hydroxyphenylpyruvate and the three cocoon performance traits (WCY, CSR and CSW). In this study, the above six preselected potential candidate biomarkers were defined as biomarkers relating to cocoon yield performance traits, which deserve further study.

## 4 Discussion

To our knowledge, this study is the first to integrate the metabolomic profiles of three tissues (MG, HL and PSG) with cocoon yield performance traits (WCY, CSW and CSR) in factory sericulture research. The current study also provides a method to systematically identify the metabolic reasons for the poor cocoon performance of the FF silkworms. We also performed pathway analysis and biomarker identification to explore the internal metabolic mechanisms and key biomarkers relating to the cocoon yield, thus providing future directions for improving the cocoon performance of the FF silkworms by optimizing the nutrition supply.

### 4.1 Poor cocoon performance of the FF silkworms

The experimental *B. mori* variety “Zhong 2016×Ri 2016” has been widely reared in a commercial enterprise. Although the CSW and CSR of the FF group were lower than those of the control ML silkworms, they also reached the average levels of the ML silkworms reported in previous studies (Qin et al., 2020; Xiao et al., 2020). Furthermore, given the high feeding adaptability to formula feed, developmental uniformity and silk quality of the silkworm variety with the relatively high efficiency of factory sericulture, feeding silkworms on formula feed has demonstrated practical application value. However, much room remains for cocoon performance improvement in the FF silkworms. Systematic metabolomic research provides a perspective to identify the internal mechanisms relating to cocoon and nutrition supply, which should aid in improving cocoon performance.

### 4.2 Significantly different metabolites

Regarding the metabolic differences between the FF and ML silkworms in three experimental tissues, we assessed, validated and classified the SDMs in the online Kyoto Encyclopedia of Genes and Genomes database (KEGG: https://www.kegg.jp/) and the Human Metabolome Database (HMDB: https://hmdb.ca/metabolites). Our analysis indicated that a large proportion of SDMs were significantly higher in the FF silkworms, a finding which was inconsistent with a previous report (Tao et al., 2022). Tao et al. (2022) has reported that the FF group had more downregulated metabolites in the HL than the ML group, thus indicating that the formula feed inhibited the metabolic activity of silkworms. This contradictory results might due to the nutrient levels of the formula feed. In our study, the WCY of FF silkworms was greater than the control group (ML group). However, in the report of Tao et al. (2022), the three cocoon performance traits of FF silkworms were much lower than the control, which might indicate the nutrient supply didn’t meet the requirement of FF silkworms.

To explore the largest differences in metabolomic profiling, the top 15 upregulated and top 15 downregulated SDMs were considered.

In the MG, N-methyltyramine, a potent stimulant of gastrin release with a role in promoting intestinal secretion and movement (Ohta et al., 2020), was significantly higher in the FF group. Consequently, we inferred that the absorbed energy and nutrients did not satisfy the cocoon requirements in the FF silkworms, thus increasing the release of N-methyltyramine and accelerating the digestion and absorption in the MG. N-methyltyramine is also involved in tyrosine metabolism (Leete and Marion, 1953). On the basis of this finding, combined with lower levels of tyramine (an intermediate metabolite from tyrosine to N-methyltyramine) in the HL, we inferred that more tyrosine was metabolized into N-methyltyramine in the FF silkworms. Furthermore, tyrosine was significantly higher in the PSG of the FF silkworms. Thus, we inferred that more tyrosine was supplied in the experimental formula feed. Tyrosine, one of the four main components of silk, might not be the limiting factor for silk synthesis in the FF silkworms. Pyridoxine 5′-phosphate was one of the top 15 downregulated SDMs in the FF silkworms, which participates in pyridoxine (vitamin B_6_) metabolism (KEGG PATHWAY: map00750). Combined with the significantly diminished pyridoxine in the FF silkworms, the above results indicated that pyridoxine was in short supply in formula feed. Further, pyridoxine 5′-phosphate is involved in cofactor biosynthesis, thus indirectly affecting several amino acids, including serine, glutamine, methionine and cysteine metabolism (KEGG PATHWAY: map01240). Thus, we inferred that insufficient pyridoxine resulted in a lower pyridoxine 5′-phosphate level, which in turn restricted the corresponding amino acid metabolism. Ultimately, the cocoon yield decreased in silkworms reared on formula feed. Moreover, pyridoxine 5′-phosphate can be converted into pyridoxal 5′-phosphate through interaction with the pyridoxine-5′-phosphate oxidase (Huang et al., 2016a; Huang et al., 2016b). Pyridoxine-5′-phosphate oxidase is regulated by development and hormones (juvenile hormone, molting hormone) in silkworms (Huang et al., 2016a). Thus, the lower pyridoxine 5′-phosphate in the FF silkworms might be related to the hormone levels-a possibility requiring further validation.

In the HL, seven of the top SDMs were classified as flavonoids and isoflavonoids, given that flavonoid and isoflavonoid metabolism differs among diet treatments. Among above seven SDMs, all flavonoids were significantly higher in the ML group, whereas all isoflavonoids were much higher in the FF silkworms. (+)-Norpseudoephedrine plays a role in suppressing appetite (Balasubramanian, 2020). Thus, the significantly higher concentration of (+)-norpseudoephedrine in the FF group might indicate lower feed intake of the FF silkworms. The substrate of riboflavin (vitamin B_2_), 6,7-dimethyl-8-(D-ribityl)lumazine (Zylberman et al., 2006), was much higher in the FF group. Correspondingly, the riboflavin level in the FF silkworms was significantly higher than that in the ML group. These results indicated that the riboflavin supply was beyond the requirement, a result warranting further validation in the FF silkworms. Tyramine, a monoamine compound derived from nondigestible tyrosine-containing peptides and proteins by bacterial fermentation (Anderegg et al., 2020), was markedly lower in the FF silkworms. Our results showed that the tyrosine to tyramine pathway was less active in the FF group, possibly because of less bacterial fermentation activity. However, this possibility requires further validation.

Among the top 30 SDMs (15 upregulated and 15 downregulated) in the PSG, 11 SDMs were classified as flavonoids and isoflavonoids, thus further validating that the flavonoid and isoflavonoid supply or metabolism markedly differed between the FF and ML groups. Among the 11 SDMs, eight were enriched in the ML group, a result consistent with the high number of flavonoids in mulberry leaves. Higher content of cysteine glutathione disulfide might imply that the FF silkworms were in a state of oxidative stress, because cysteine glutathione disulfide is a product of glutathione oxidation (Eriksson et al., 1967). Thus, the health and immune status of the FF silkworms should be examined with biochemical methods in the future.

A total of 60 mutual SDMs were identified in the three experimental tissues. Among them, 48 up-regulated and two down-regulated SDMs showed the same difference patterns. In silkworm larvae, superfluous amino acids are deaminized in the fat body and MG, thus producing ammonia, which is removed in three forms (Weihrauch and O'Donnell, 2021). Part of the ammonia is excreted in the form of ammonium salt by the Malpighian tubules (Weihrauch and O'Donnell, 2021), and a portion of the ammonia is stored as amides to provide amidogen for amino acid synthesis (Hirayama et al., 1997), or is involved in purine synthesis and eventually metabolized to uric acid or urea (Hirayama et al., 1996; Hiroko et al., 2016). Uric acid and urea, the end-product of amino acid metabolism, were significantly higher in all three tissues in the FF group than the ML group, possibly because of the higher urease activity of mulberry leaves (Hirayama et al., 1999). We speculated that urease was inactivated in the production and heat treatment of formula feed, thus impairing the metabolism of proteins, amino acids and other nitrogen-containing substances, which was consistent with a previous report (Tao et al., 2022). Many amino acids or small peptides including histidine, asparagine, homocitrulline and gamma-glutamylleucine were enriched in the FF tissues, in agreement with the above hypothesis. The difference trends for ascorbic acid were discrepant in three tissues: the levels were markedly lower in the MG and HL of the FF silkworms, but higher in the PSG. Ascorbic acid, also called vitamin C, is a crucial nutrient for the growth and development of silkworm larvae (Sayyad, 2020) and is obtained mainly from the diet. Thus, we inferred that the ascorbic acid supply was insufficient in the FF silkworm larvae, thus restricting the development of silkworms. The discrepant patterns of ascorbic acid in different tissues might be related to ascorbic acid metabolism, as observed for ascorbate and aldarate metabolism in the metabolic pathways enriched in the HL and PSG.

### 4.3 Metabolic pathways

In this study, the SDMs of different diets were found to be classified and involved mainly in the metabolism of amino acids and other nitrogen-contained intermediates, lipids, carbohydrates, vitamins, flavonoids and isoflavonoids. Some studies have found that insufficient vitamin supply; higher urea and uric acid content; and metabolic disorders related to amino acid, carbohydrate and lipid metabolism are the main discrepancies between silkworms fed ML and FF (Dong et al., 2017; Qin et al., 2020; Tao et al., 2022). To identify crucial pathways relating to the diet treatments, we put the mutually enriched pathways among three tissues to the first place. The PSG serves as the production locus for fibroin, a main component of silk (Shigematsu and Koyasako, 1962). Besides, the SDM amounts in the PSG were much higher than those in the MG and HL, thus illustrating that the metabolomic differences affected by the rearing method were highest in the PSG; therefore, our analysis was focused on the top three mutual pathways with the highest IPV in the PSG.

With the highest IPV, cysteine and methionine metabolism was enriched by ten SDMs in the PSG. The ten SDMs accounted for approximately one-third of the entire pathway. Notably, the log_2_FC values revealed that all SDMs involved in cysteine and methionine metabolism were significantly higher in the FF group. Cysteine is derived from the diet or biosynthesis, with the latter source used serine as the substrate (KEGG Module: M00021, M00338). The serine content was indeed higher in the PSG of the FF silkworms. Serine is one of the four main components of silk (Kensuke et al., 1976). Given the biological function of the PSG, our results might indicate that the higher serine was not integrated into silk as expected but was metabolized into cysteine or other intermediates. This possibility is supported by the higher content of L-cystathionine, 2-oxobutanoate and L-homocysteine, which are intermediate products of serine metabolism in the cysteine and methionine metabolism pathway (KEGG PATHWAY: map00270). Thus, the above possibility might partly explain the contradictory finding of higher serine and lower silk performance traits in the FF silkworms. Methionine has been reported to be upregulated in silkworms reared on formula feed (Tao et al., 2022). As an essential amino acid, methionine cannot be synthesized by silkworms (Ito, 1967). Thus, we inferred that the higher content of methionine might have been derived from the diet or bacterial synthesis.

Arginine biosynthesis (KEGG PATHWAY: map00220), with an impact value of 0.629, was the 2nd mutual metabolic pathway enriched in the three experimental tissues. Arginine, an essential amino acid for silkworms, has been speculated to be synthesized mainly by the microbiota (Ito, 1967). On the one hand, the content of L-glutamate, N-acetylornithine and L-citrulline, which are substrates for arginine synthesis (KEGG Module: M00845), were all higher than those in the ML group. On the other hand, three metabolic products of arginine had higher levels in the FF silkworms: L-ornithine, L-citrulline and urea. Consequently, we speculated that the above arginine pathways involved in synthesis and metabolism might be more active in the FF silkworms. As noted in Discussion section: Significantly different metabolites, the substantially high FC of urea (Log_2_FC = 5.43) implied that the free arginine exceeded the requirements for normal growth and development of the FF silkworms. Further, combined with the lack of urease, the higher content of urea could not be hydrolyzed into ammonia to provide nitrogen for silkworm growth and development. Thus, the higher content of arginine might be detrimental indirectly for the silkworm growth and development.

Arginine and proline metabolism, with the 3rd highest IPV, was discussed subsequently. Arginine and proline are essential and semi-essential amino acids for silkworms (Ito, 1967), respectively. In the current study, the metabolic products of arginine, including L-ornithine, putrescine and L-glutamate (KEGG PATHWAY: map00330), all showed higher content in the FF silkworms. Although L-proline was not identified as an SDM in the PSG, owing to its low VIP value (VIP <1.00), it was numerically higher in the FF silkworms (FDR <0.05). Proline can be transformed by arginine and ornithine (Xu et al., 1980) to partly or completely meet the requirement of silkworm growth. In the FF group, proline was significantly higher in the MG, which indicated the high content of proline was partly derived from the diet source. The lack of proline could restricted the development and growth of silkworms (Ito, 1972). In this study, the higher content of proline in the FF group might be helpful to explain the heavier pupae weight of the FF group. The higher proline content was metabolized into hydroxyproline and L-glutamate, both of which were significantly higher than those in the ML silkworms (*p* < 0.05). L-glutamate is an essential amino acid for silkworm (Ito, 1972), which provides amidogen in transaminatin. Under the role of alanine aminotransferase, L-glutamate could be metabolized into pyruvic acid (Fukuda, 1957), which could provide energy for silk synthesis. However, pyruvic acid was not significantly different between the FF and ML silkworms, which might be attributed to the less active alanine aminotransferase in the FF silkworms. It was reported alanine aminotransferase was less active when the vitamin B_6_ was insufficient in silkworms (Horie and Nakamura, 1986). In this study, we inferred vitamin B_6_ was in short supply in the Discussion section: Significantly different metabolites. Thus, we thought the insufficient vitamin B_6_ restricted the activity of alanine aminotransferase which further limited the transformation of L-glutamate to pyruvic acid and restricted the silk synthesis ultimately.

### 4.4 Biomarkers associated with cocoon yield performance traits

Biomarker analysis combined with a Pearson correlation calculation revealed six biomarkers that showed positive or negative relationships with the WCY, CSW and CSR: homocitrulline (Log_2_FC = 4.88, 4.82 and 8.88 in the MG, HL and PSG, respectively), glycitein (Log_2_FC = 12.6, 9.83 and 8.43 in the MG, HL and PSG, respectively), valyl-threonine (Log_2_FC = 4.56, 4.28 and 5.19 in the MG, HL and PSG, respectively), propyl gallate (Log_2_FC = 12.1, 9.70 and 14.3 in the MG, HL and PSG, respectively), 3-amino-2,3-dihydrobenzoic acid (Log_2_FC = 1.93, 2.44 and 3.97 in the MG, HL and PSG, respectively) and 3-dimethylallyl-4-hydroxyphenylpyruvate (Log_2_FC = -2.98, -3.64 and -4.60 in the MG, HL and PSG, respectively). Among these six biomarkers identified, the first five showed a positive relationship with WCY, and a negative relationship with CSW and CSR.

Homocitrulline, an L-alpha amino acid, is a post-translational modification product (Mahmoudi et al., 2019). As reported, homocitrulline is closely associated with inflammatory responses, physical condition and diseases (Lac et al., 2018), and has been proposed to serve as a biomarker for identifying patient frailty (Mahmoudi et al., 2019) or oxidative stress relating to myeloperoxidase (Pireaux et al., 2021). In this study, homocitrulline was significantly higher in the FF silkworms, particularly in the PSG (Log_2_FC = 8.88). Thus, we speculated that the FF silkworms might have been in a state of oxidative stress or weakness, a possibility warranting further exploration.

Glycitein, an isoflavonoid, was detected only in the FF group in this experiment. It is present in high concentrations in natto, miso and soybeans, according to the HMDB (https://hmdb.ca/metabolites/HMDB0005781). Therefore, our results were reasonable, given that soybean meal was a major component of the experimental formula feed (1^st^ to 3^rd^ instar silkworm feed: 31%, 4th and 5th instar silkworm feed: 40%). Moreover, glycitein, a primary metabolite, is directly associated with the growth, development and reproduction of organisms (Hu et al., 2015; Pastore et al., 2018). The extremely high glycitein might contribute to greater growth and development of silkworm larvae, thus resulting in a positive correlation between glycitein and pupa weight, as included in the WCY.

Valyl-threonine, a dipeptide of valine and threonine, is an incomplete breakdown product in protein digestion or catabolism (https://hmdb.ca/metabolites/HMDB0029137). The high content of valyl-threonine might have indicated incomplete proteolysis in the FF silkworms, thereby restricting the substrate supply for silk synthesis. Thus, this compound was negatively correlated with CSR and CSW. Moreover, some dipeptides have been found to be directly used in organism development (Fedoreyeva et al., 2020), thus possibly explaining the positive relationship with WCY.

Propyl gallate, an antioxidant used in the food industry (Nguyen et al., 2021), was significantly higher in the FF group. Propyl gallate is found in corn, a major component of the experimental formula feed. In addition, gallic acid, a part of propyl gallate, was added in the formula feed as a preservative. Thus, the content of propyl gallate was affected by the diet treatment. Propyl gallate is absorbed after ingestion (Becker, 2007), and thus showed a relatively higher concentration in the MG, which plays important roles in digestion and absorption (Shen et al., 2022). The HL, a circulatory system for nutrients and metabolites, also contained more propyl gallate in the FF group. The biological roles of propyl gallate include scavenging of oxygen free radicals, antimicrobial activity, inhibitory enzyme activity and biosynthetic processes inhibition (Becker, 2007). Thus, the higher concentration of propyl gallate might have resulted in lower microbiota and enzymatic activity, thereby restricting nutrient metabolism and silk synthesis in the FF group. Furthermore, propyl gallate shows slight toxicity when ingested (Becker, 2007) and therefore might have influenced silkworm health status.

The compound 3-amino-2,3-dihydrobenzoic acid, in the category of amino acids, peptides and analogues, showed higher concentrations in all experimental tissues of the FF silkworms. This compound, also known as gabaculin or 5-aminocyclohexa-1,3-diene-1-carboxylic acid, exists in all living organisms and inhibits the activity of γ-aminobutanoic acid-transaminase (Park et al., 2021) and ornithine aminotransferase (Li et al., 2016). Inhibition of ornithine aminotransferase results in restriction of ornithine synthesis with glutamate (KEGG Module: M00028) as a substrate. Thus, the higher content of 3-amino-2,3-dihydrobenzoic acid might have restricted the corresponding amino acid metabolism in the FF silkworms and partially explained the negative correlations between 3-amino-2,3-dihydrobenzoic acid and CSW, and 3-amino-2,3-dihydrobenzoic acid and CSR. In plant research, 3-amino-2,3-dihydrobenzoic acid is used as an inhibitor of chlorophyll biosynthesis, a tetrapyrrole synthesis relating to heme and holocytochrome (Demko et al., 2010).

As the only biomarker which was negatively correlated with WCY and positively correlated with CSW and CSR, 3-dimethylallyl-4-hydroxyphenylpyruvate was significantly lower in the FF silkworms. Of note, 3-dimethylallyl-4-hydroxyphenylpyruvate was also the only top SDM identified mutully in all experimental tissues in this research. On the basis of the KEGG database, 3-dimethylallyl-4-hydroxyphenylpyruvate is involved in novobiocin biosynthesis (KEGG PATHWAY: map00401) and biosynthesis of secondary metabolites (KEGG PATHWAY: map01110). In the novobiocin biosynthesis pathway, 3-dimethylallyl-4-hydroxyphenylpyruvate is indirectly synthesized by L-tyrosine. Given the higher tyrosine and lower 3-dimethylallyl-4-hydroxyphenylpyruvate levels, we inferred that the novobiocin biosynthesis pathway was less active in the FF silkworms.

## 5 Conclusion

In this study, systematic metabolomic profiles of the MG, HL and PSG were integrated to clarify the metabolic characteristics of silkworms fed formula feed versus mulberry leaves. Cysteine and methionine metabolism; arginine biosynthesis; and arginine and proline metabolism were mutual key pathways affecting cocoon yield performance traits. Homocitrulline, glycitein, valyl-threonine, propyl gallate, 3-amino-2,3-dihydrobenzoic acid were positively correlated with WCY, but negatively correlated with CSW and CSR, while 3-dimethylallyl-4-hydroxyphenylpyruvate showed an opposite correlation pattern. In other words, the lower level of homocitrulline, glycitein, valyl-threonine, propyl gallate, 3-amino-2,3-dihydrobenzoic acid and the higher level of 3-dimethylallyl-4-hydroxyphenylpyruvate, the higher CSW and CSR might be achieved, which requires further validation by a nutritional experiment further.

## Data Availability

The original contributions presented in the study are included in the article/Supplementary Material, further inquiries can be directed to the corresponding author.

## Abbreviations

CSW, Cocoon shell weight; CSR, Cocoon shell rate; ESI, Electrospray ionization; FF, Formula feed; HL, Hemolymph; IPV, Impact value; MG, Midgut; ML, Mulberry leaves; MVDA, Multivariate data analysis; PSG, Posterior silk gland; QC, Quality control; QQQ, Triple quadrupole; SDM, Significantly different metabolite; WCY, Whole cocoon yield.
