# Supplementary figures and images for: Multi-tissue metabolomic profiling reveals potential mechanisms of cocoon yield in silkworms (Bombyx mori) fed formula feed versus mulberry leaves
Source: Front Mol Biosci. 2022 Aug 17;9:977047. doi: 10.3389/fmolb.2022.977047 (PMC9428324; doi:10.3389/fmolb.2022.977047)

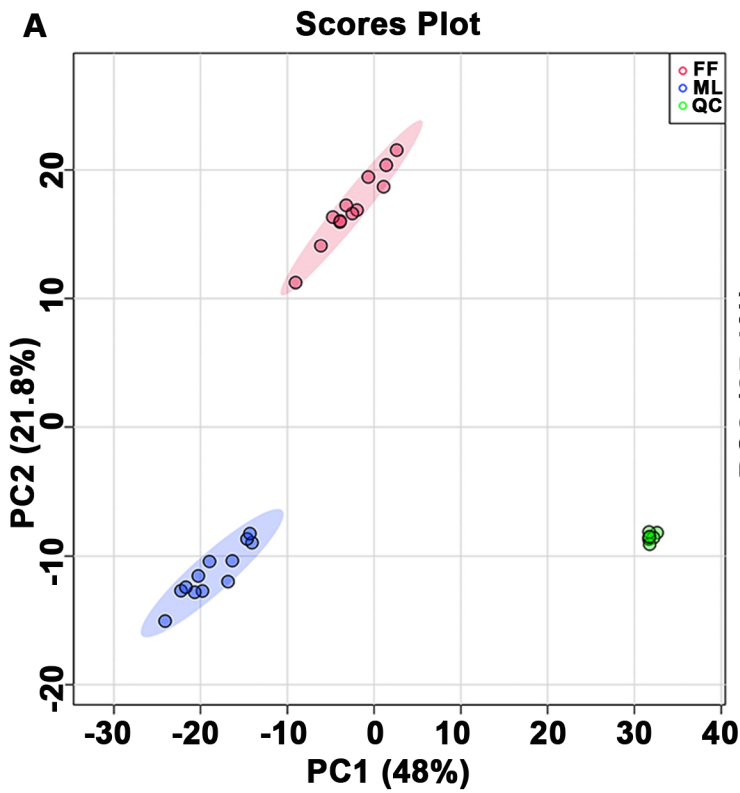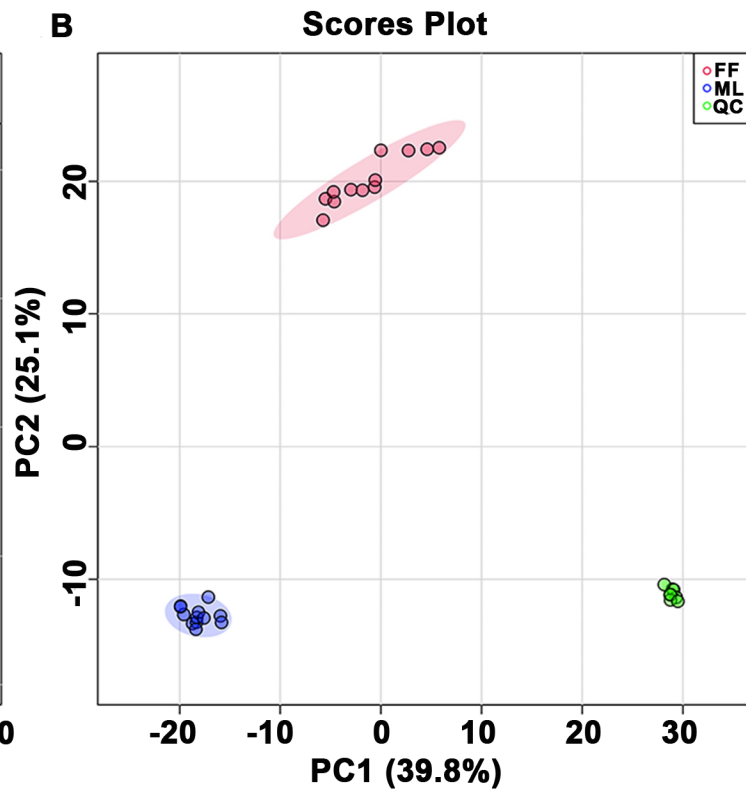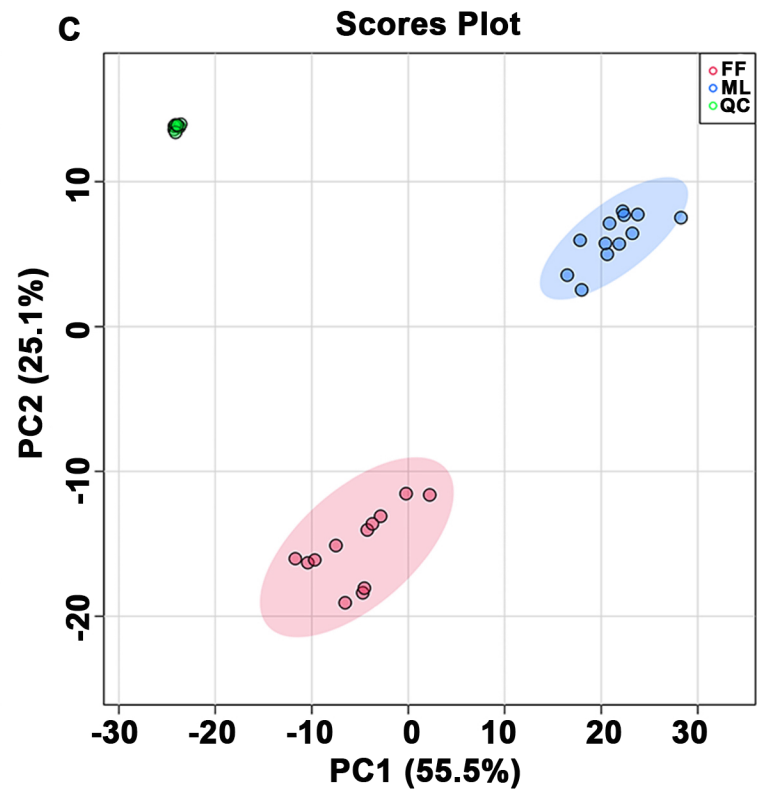

Supplement: Supplementary file 1 [file Image2.pdf]

**Hemolymph**

**Midgut**

**116**

**34**

**75**

**60**

**39**

**61**

**144**

**Posterior silk gland**

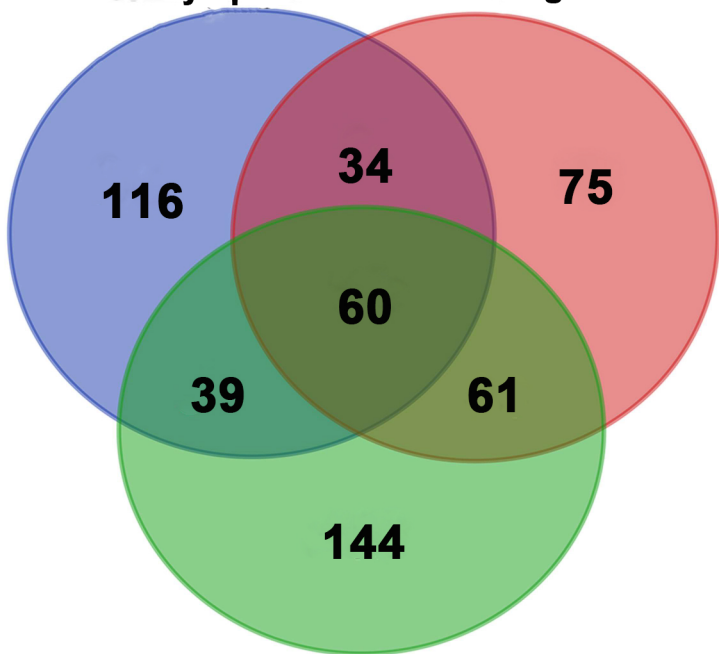

Supplement: Supplementary file 2 [file Image3.PDF]

**A**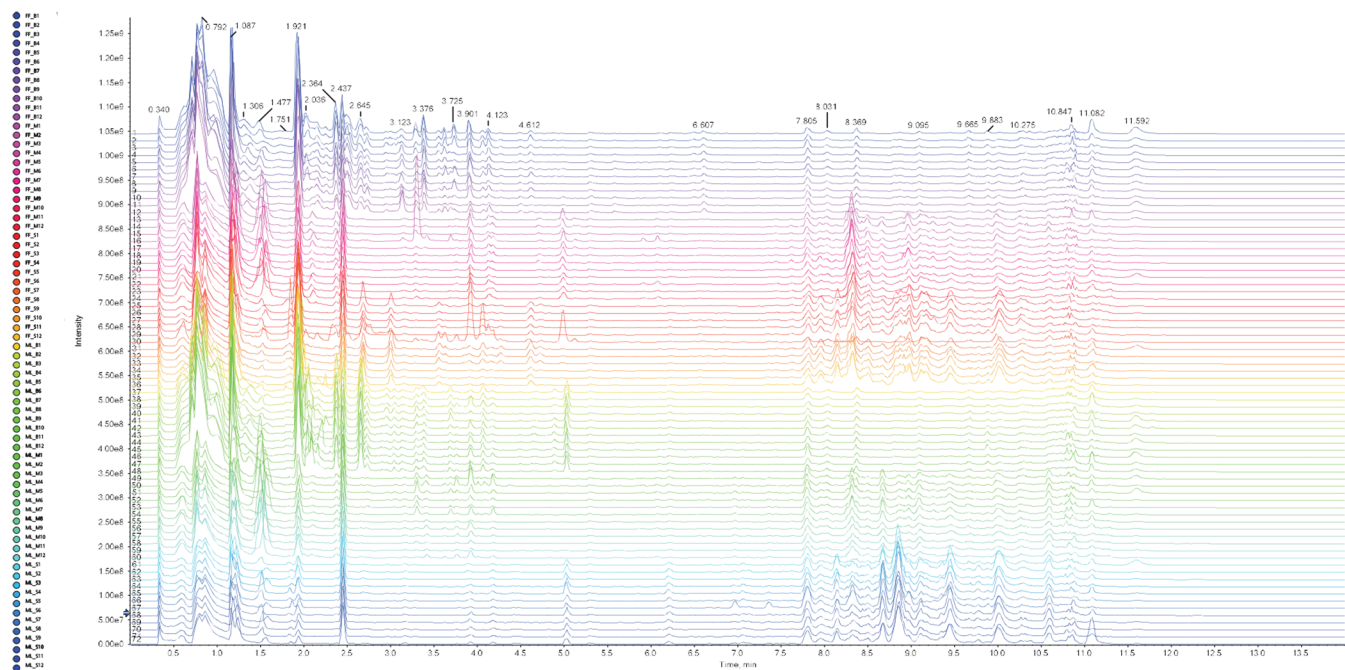**B**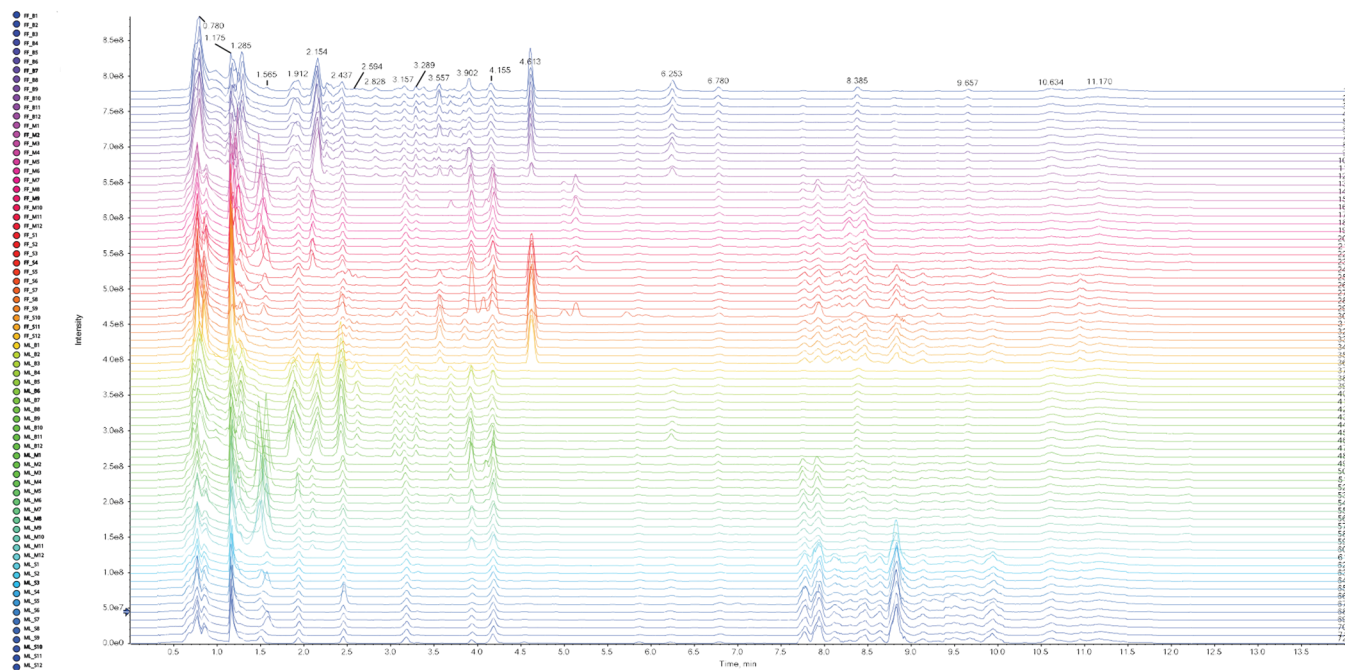

Supplement: Supplementary file 3 [file Image1.PDF]
